# CA125 Levels in BRCA mutation carriers – a retrospective single center cohort study

**DOI:** 10.1186/s12885-023-11116-6

**Published:** 2023-07-01

**Authors:** P Gebhart, CF Singer, D Gschwantler-Kaulich

**Affiliations:** 1 Department of Obstetrics and Gynaecology, Comprehensive Cancer Center, Medical University of Vienna, Vienna, Austria; 2 Department of Obstetrics and Gynecology, University Hospital Vienna, Währinger Gürtel 18-20, 1090 Vienna, Austria

**Keywords:** CA-125 antigen, Genes, BRCA1, Genes, BRCA2, Hereditary breast and ovarian cancer syndrome, Early detection of cancer, Ovarian neoplasms

## Abstract

**Background:**

Ovarian cancer screening in BRCA1/2 mutation carriers utilizes assessment of carbohydrate antigen 125 (CA125) and transvaginal ultrasound (TVU), despite low sensitivity and specificity. We evaluated the association between CA125 levels, BRCA1/2 mutation status and menopausal status to provide more information on clinical conditions that may influence CA125 levels.

**Methods:**

We retrospectively analyzed repeated measurements of CA125 levels and clinical data of 466 women at high risk for ovarian cancer. CA125 levels were compared between women with and without deleterious mutations in BRCA1/2. Pearson's correlation was used to determine the association between age and CA125 serum level. Differences in CA125 levels were assessed with the Mann–Whitney U test. The effect of BRCA1/2 mutation status and menopausal status on the change in CA125 levels was determined by Two-factor analysis of variance (ANOVA).

**Results:**

The CA125 serum levels of premenopausal women (median, 13.8 kU/mL; range, 9.4 – 19.5 kU/mL) were significantly higher than in postmenopausal women (median, 10.4 kU/mL; range, 7.7 – 14.0 kU/mL; *p* < .001). There was no significant difference in the CA125 levels of BRCA mutation carriers and non-mutation carriers across all age groups (*p* = .612). When investigating the combined effect of BRCA1/2 mutation and menopausal status, variance analysis revealed a significant interaction between BRCA1/2 mutation status and menopausal status on CA125 levels (*p* < .001). There was a significant difference between the CA125 levels of premenopausal and postmenopausal women, with a large effect in BRCA mutation carriers (*p* < .001, d = 1.05), whereas in non-mutation carriers there was only a small effect (*p* < .001, d = 0.32).

**Conclusion:**

Our findings suggest that hereditary mutations in BRCA1/2 affect the decline of CA125 levels with increasing age. To prove a definite effect of this mutation on the CA125 level, prospective trials need to be conducted to define new cut-off levels of CA 125 in mutation carriers and optimize ovarian cancer screening.

## Background

Epithelial ovarian cancer is the most lethal gynecological cancer with an overall survival of 46% after diagnosis. The poor prognosis is mainly caused by the usually advanced stage of disease at the time of diagnosis due to the asymptomatic nature of ovarian cancer (OC) [1]. Women considered to be at “high-risk” for OC typically have a significant family history for OC as well as breast cancer and carry pathogenic mutations in genes that suppress oncogenesis of these diseases. Germline mutations in the BRCA1 and BRCA2 genes are associated with a high risk for OC and cause about 15–20% of all cases. The cumulative lifetime risk for OC by age 80 amounts to 44% in BRCA1-mutation carriers and 17% in BRCA2-mutation carriers [2].

For women at high-risk with BRCA1/2 mutations, risk-reducing salpingo-oophorectomy (RRSO) is the only proven mortality reducing intervention to prevent OC or fallopian tube cancer and to detect occult neoplasia. It is recommended for women older than 35 years who have already completed childbearing [3, 4]. Although RRSO is very effective when performed premenopausal [4], it causes infertility and premature menopause [5], which is associated with an increased risk for osteoporosis [6] as well as cardiovascular [7] and neurologic disease [8]. Some female BRCA mutation carriers defer RRSO until menopause or decline the intervention altogether, despite the risks. These women would especially profit from an effective OC screening strategy [9, 10].

The current screening for high-risk women includes tumor marker testing for serum CA125 and transvaginal ultrasound (TVU) every 6 months [11]. However, the performance of these tests has been poor, as sensitivity and specificity of CA125 and TVU are not sufficient for screening [12–14]. The measurement of CA125 for early detection of OC is limited by false-negative results, due to the low sensitivity in early-stage disease, as well as false-positive results occurring from elevation in physiological and benign conditions [15, 16]. These include gynaecologic conditions like endometriosis as well as non-gynaecologic disorders such as liver disease or pancreatitis [17–20]. Demographic and clinical factors like race, age and the intake of oral contraceptives have also been shown to affect CA125 serum levels [21–23].

CA125 serum levels are connected to ovarian function and decrease with age [16, 21], which results in significantly higher values in premenopausal than in postmenopausal women [24, 25]. Therefore, for postmenopausal women, a cut-off of 35 kU/mL is recommended, whereas in premenopausal women a cut-off of 50 kU/mL is used [22]. While the current detection of OC relies on the follow-up of CA125 levels above the mentioned cut-offs, a rising level over time within the normal limits has also been shown to be a sign for the development of this disease [10, 26, 27].

The association between ovarian cancer and increased CA125 serum levels is stronger in postmenopausal than in premenopausal women [28]. Many studies on serial CA125 measurements have been conducted on postmenopausal women in average-risk populations [17, 21, 27, 29, 30]. However, in most women at high hereditary risk for ovarian cancer, screening begins before menopause [31]. The current OC screening regimens have not been able to provide a mortality reduction in the average population as well as in high-risk patients [10, 12, 27]. An improvement in the early detection of ovarian cancer is highly needed, especially for women at high hereditary risk.

The aim of this study was to investigate whether there is a difference in CA125 concentrations in women with a mutation in the BRCA1 or BRCA2 gene and women without this genetic mutation and to analyze changing concentrations depending on age and menopausal status.

## Materials and methods

### Study population

This retrospective analysis is based on the data of women participating in the high-risk early detection program for hereditary breast and ovarian cancer (HBOC) of the Department of Gynecological Oncology of the Medical University of Vienna between 2000 and 2018. This cohort included women who were considered to be at an elevated risk for breast and ovarian cancer due to a significant family history and/or mutations in BRCA1/2. We compared the data of women with a deleterious BRCA1/2 mutation to those without a deleterious BRCA1/2 mutation. Data regarding CA125 serum levels, BRCA1 or BRCA2 mutation status, age, menopausal status, RRSO and ovarian cancer were collected by retrospective chart review.

The study included women without ovarian cancer, who had been tested for mutations in BRCA1 or BRCA2 and had not yet undergone RRSO. Women diagnosed with ovarian cancer, unknown BRCA1/2 mutation status or no CA125 measurements were excluded. Women with clinical chart information specifically stating the presence of endometriosis or pelvic inflammatory disease were also excluded. There was a considerable difference in the number of CA125 measurements per patient. Hence, we evaluated each measured CA125 value as its own case. We were not able to retrospectively assess most clinical factors which can significantly influence CA125 in healthy women. This led to a substantial variety of CA125 values in our collected data with many values which surpassed the threshold of 35 kU/L or 50 kU/L by far. Clinical studies evaluating CA125 serum concentrations in women with stage III/IV endometriosis, endometrioma or pelvic inflammatory disease have predominantly reported mean CA125 levels ranging between 60 – 70 kU/L [32–36]. Based on this data, we assumed that in non-malignant cases with CA125 values greater than 70 kU/L, the presence of a benign gynecological disorder was extremely high. Consequently, we excluded these cases from our analysis.

Considering our inclusion and exclusion criteria, we were able to use the data of 466 women with overall 1305 CA125 measurements in our analyses. Population based studies have shown that natural menopause usually occurs at a median age of 51 years in high-income countries [37, 38]. Therefore, we defined cases ≥ 51 years and older as postmenopausal for patients in whom no information on menstrual history was available, unless there was specific clinical chart information available stating that the patient was still premenopausal at the time of screening. Otherwise, women aged < 51 years were classified as premenopausal. The characteristics of the patients are summarized in Tables 1 and 2.

**Table 1:** Characteristics of Women at High Hereditary Risk at First Visit

	BRCA1/2 Mutation		Total
	Carriers^a^	No mutation^b^	
Age, years			
Median	41	45	45,1
Range	26—73	24—77	24—77
Menopausal status			
Premenopausal	103	235	338
% within all Premenopausal	30,5%	69,5%	
% of Total	22,1%	50,4%	72,5%
Postmenopausal	29	99	128
% within all Postmenopausal	22,7%	77,3%	
% of Total	6,2%	21,2%	27,5%
Total	132 (28,3%)	334 (71,7%)	466

^a^Carriers refers to women with a deleterious mutation in the BRCA1 or 2 genes

^b^No mutation refers to women without a deleterious mutation in the BRCA1 or 2 genes

**Table 2:** Distribution by age and menopausal status of high-risk women at each measurement

	BRCA1/2 Mutation		Total
	Carriers^a^	No mutation^b^	
Age, years			
Median	49	44	47,5
Range	26—80	24—83	24 – 83
Menopausal status			
Premenopausal	496 (38,1%)	357 (27,4%)	853 (65,4%)
Postmenopausal	343 (26,2%)	109 (8,3%)	452 (34,6%)
Total	839 (64,3%)	466 (35,7%)	1305 (100%)

^a^Carriers refers to women with a deleterious mutation in the BRCA1 or 2 genes

^b^No mutation refers to women without a deleterious mutation in the BRCA1 or 2 genes

## Statistical analysis

Statistical analysis was performed using SPSS software (Software IBM SPSS® 26.0). The strength of the relationship between age and CA125 serum level was determined by Pearson correlation coefficient (r), provided that the two variables were in linear association. Additionally, we calculated the confidence intervals for the correlation coefficient r by bootstrapping, estimating the true mean of the sample with a probability of 95%. Bootstrapping was performed by case resampling. Differences in CA-125 levels were assessed with the Mann–Whitney U test. The effect of BRCA1/2 mutation status and menopausal status on the change in CA125 levels was determined by Two-factor analysis of variance (ANOVA). Levene’s test was performed to verify the homogeneity of variances. To confirm where the differences occurred between groups, post hoc Welch-t-test was performed. Two-sided testing was applied, p-values below 0.05 were considered statistically significant. No adjustment for multiple testing has been performed, as the aims of this study are exploratory. To evaluate the extent of the differences of the results, effect size d according to Cohen was used, which suggests that d ≥ 0.20 is a small, d ≥ 0.50 a medium and d ≥ 0.80 a large effect size. Correlation coefficient r was interpreted as effect size for correlation, with values of *r* ≥ 0.10 representing a small, *r* ≥ 0.30 a medium and *r* ≥ 0.50 a large effect size.

## Results

This analysis is based on 466 women among whom 1305 evaluations of the CA125 serum concentration were conducted (Tables 1 and 2). The median age at first visit was 45.1 years (min 24.2, max 77.6) years (Table 1). The average number of measurements per patient was 2,83 (SD = 3,33; min 1, max 38).

### CA125 Levels depending on age

Regardless of mutation status, there was a small but significant negative correlation between age and CA125 serum level, r(1305) = -0.22; 95%-KI [-0.17; -0.28], *p* < 0.001. Applying the associated regression equation, Ŷ = 23.49 + (-0.182 * x_i_), it can be assumed that the CA125 serum level of a 50-year-old woman is measured at 14.39 kU/l, independent of mutation status. Considering mutation status, there was a very small but significant negative correlation between age and CA125 serum level, r(839) = -0.12; 95%-KI [-0.05; -0.19], *p* < 0.001, in cases with no mutation in BRCA1/2. Using the associated correlation equation, Ŷ = 19.55 + (-0.104 * x_i_), the CA125 serum level of a 50-year-old non-mutation-carrier can be assumed at 14.35 kU/l. For cases with a BRCA1/2 mutation, a moderate negative correlation was found, r(466) = -0.34; 95%-KI [-0.27; -0.42], *p* < 0.001. On the base of the associated correlation equation, Ŷ = 28.28 + (-0.288 * x_i_), a CA125 serum level of 13.88 kU/l can be assumed for a 50-year-old BRCA1/2—mutation carrier.

### CA125 Levels in BRCA1/2 mutation carriers and non-mutation carriers

CA125 levels were higher (*p* < 0.001) in premenopausal (median, 13.8 kU/mL; range, 9.4 to 19.5 kU/mL) than in postmenopausal women (median, 10.4 kU/mL; range, 7.7 to 14.0 kU/mL). CA125 levels are listed in Table 3.

**Table 3:** CA125 levels in Association with Menopausal Status as well as BRCA-Mutation-Status, separately analyzed

	n	*M* ± *SD*	min—max	*Md*	IQR	Mean Rank
Menopausal Status						
Premenopausal	853	15.93 ± 9.32	2.70—67.70	13.9	9.5; 19.5	717.64
Postmenopausal	452	12.11 ± 7.26	0.60—66.50	10.5	7.9; 14.0	531.02
Total	1305	14.61 ± 8.85	0.60—67.70	12.0	8.8; 18.1	
BRCA-mutation status						
Negative^a^	839	14.31 ± 8.33	2.10 – 66.50	12.0	8.9; 17.7	649.05
Positive^b^	466	15.16 ± 9.71	0.60 – 67.70	12.75	8.7; 18.8	660.11
Total	1305	14.61 ± 8.86	0.60—67.70	12.0	8.8; 18.1	

*Abbreviations*: *n* Number, *M* Mean, *SD* Standard deviation, *min* Minimum, *max* Maximum, *Md* Median, *IQR* Interquartile range

^a^Negative refers to women without a deleterious mutation in the BRCA1 or 2 genes

^b^Positive refers to women without a deleterious mutation in the BRCA1 or 2 genes

**Fig. 1:**
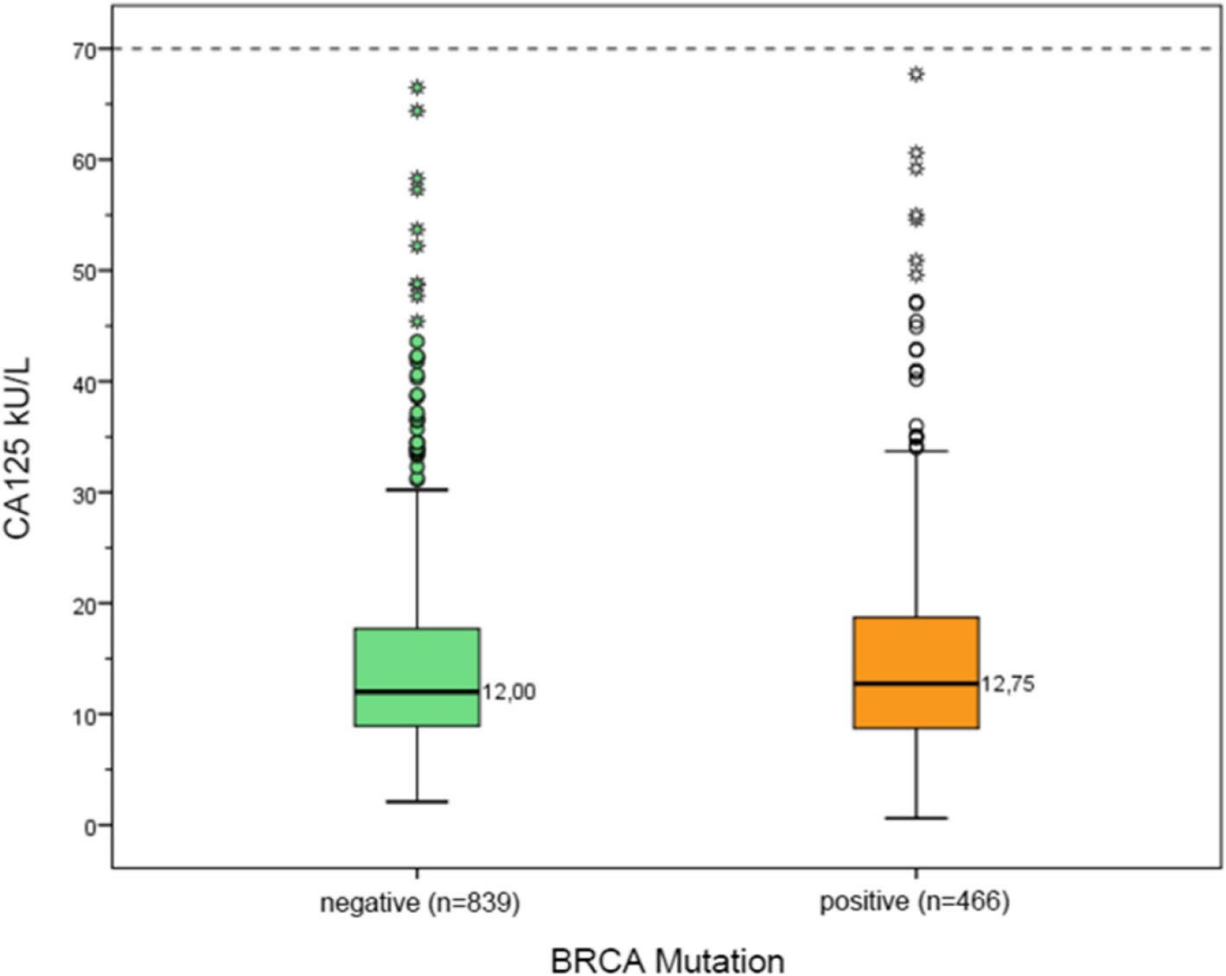
Differences in CA 125 levels (Md) between women with positive and negative BRCA-Mutation-Status. Legend: Results of the Mann–Whitney U test displayed in boxplots show no significant difference (*p* = .612) in the CA125 levels of BRCA1/2 mutation carriers (positive, orange boxplot) and non-mutation carriers (negative, green boxplot). The absolute value of CA125 in kU/mL is plotted on the y-axis. Abbreviations: (Md, Median)

We found no significant difference (*p* = 0.612) in the CA125 levels of BRCA1/2 mutation carriers (median, 12.75 kU/mL; range, 8.7 to 18.8 kU/mL) compared to non-mutation carriers (median, 12 kU/mL; range, 8.9 to 17.7kU/mL) (see Fig. 1).

### Interaction of BRCA1/2 mutation status and menopausal status

Due to the large effect of menopausal status on CA125 levels, a two-factor ANOVA was conducted to analyze the effect of BRCA1/2 mutation status and menopausal status on CA125 levels. The calculated parameters are summarized in Table 4.

**Table 4:** CA125 Serum Levels depending on BRCA-Mutation-Status and Menopausal Status

	Premenopausal			Postmenopausal			Total		
BRCA mutation status	*M*	± *SD*	n	*M*	± *SD*	n	*M*	± *SD*	n
Negative^a^	15.38	8.50	496	12.76	7.85	343	14.31	8.33	839
*Md* (IQR)	13.35	(9.63; 18.84)		10.80	(8.30; 14.90)		12.00	(8.9; 17.7)	
Positive^b^	16.72	10.34	357	10.06	4.42	109	15.16	9.71	466
*Md* (IQR)	14.20	(9.15; 21.75)		9.40	(6.80; 12.35)		12.75	(8.7; 18.75)	
Total	15.94	9.33	853	12.11	7.26	452	14.61	8.86	1305
*Md* (IQR)	13.90	(9.45; 19.50)		10.50	(7.93; 14.00)		12.00	(8.8; 18.1)	

*Abbreviations*: *n* Number, *M* Mean, *SD* Standard deviation, *min* Minimum, *max* Maximum, *Md* Median, *IQR* Interquartile range

^a^Negative refers to women without a deleterious mutation in the BRCA1 or 2 genes

^b^Positive refers to women without a deleterious mutation in the BRCA1 or 2 genes

**Fig. 2:**
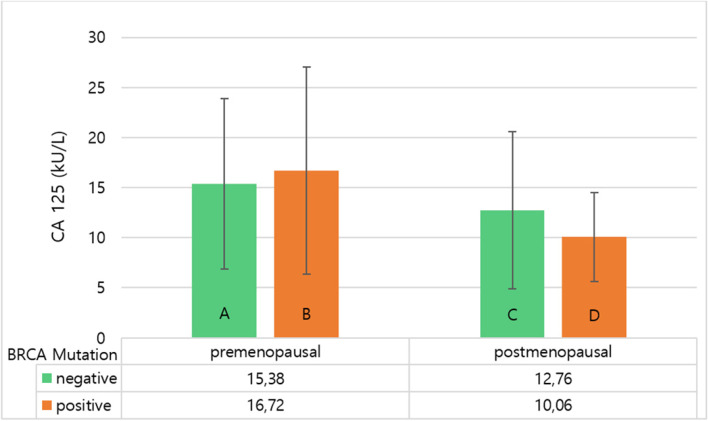
CA 125 levels (M ± 1 SD) considering BRCA-Mutation-Status and Menopausal Status. Legend: Bar graphs showing results of two-way ANOVA. **A** Showing mean CA125 values for premenopausal non-mutation-carriers (*n* = 496). **B** Showing mean CA125 values for premenopausal BRCA-mutation carriers (*n* = 357). **C** Showing mean CA125 values for postmenopausal non-mutation-carriers (*n* = 343). **D** Showing mean CA125 values for postmenopausal BRCA-mutation carriers (*n* = 109). Abbreviations: ANOVA, analysis of variance; M, mean; SD, standard deviation

There was a statistically significant interaction between the effects of BRCA1/2 mutation status and menopausal status on CA125 serum level, F(1, 1301) = 12.986, *p* < 0.001. Welch’s t-test showed that CA125 levels in non- mutation-carriers were significantly higher in premenopausal women than in postmenopausal women, t(770.98) = 4.583, *p* < 0.001 with a small effect, d = 0.32, 95% CI [0.18 – 0.46]. For BRCA1/2 mutation carriers, we also found significantly higher CA125 levels in premenopausal women compared to postmenopausal women, t(417.23) = 9.628, *p* < 0.001, but with a large effect, d = 1.05, 95% CI [0.83 – 1.28]. Figure 2 shows the mean CA125 levels by BRCA-mutation-status and menopausal status.

## Discussion

In our study, we found significantly higher CA125 levels in premenopausal women than in postmenopausal women overall. We observed decreasing CA125 levels with increasing age in both BRCA-mutation carriers and non-BRCA-mutation carriers. There was a small but significant negative correlation between age and CA125 level, regardless of mutation status. This observation is consistent with current literature [21, 22, 31]. According to our regression equation, it can be assumed that the CA125 serum level of a 50-year-old woman is measured at 14.39 kU/l, independent of mutation status. Similar results were obtained by Pauler et al., who found that CA125 levels typically lie between 13 and 22 units/ml for women who are 50 years of age [21]. Interestingly, when mutation status was considered, we observed a very small but significant negative correlation between age and CA125 level in cases with no mutation in BRCA1/2, compared to a moderate negative correlation for cases with a mutation in BRCA1/2.

In a recent study by Gschwantler-Kaulich et al. [39], significantly higher CA125 levels were observed in women with a mutation in BRCA1, compared to non-mutation carriers. We found no significant difference in the CA125 levels of BRCA1/2 mutation carriers compared to non-mutation carriers overall. Hermsen et al. likewise reported no significant difference in CA125 levels of women at high hereditary risk, compared to healthy controls [31]. A retrospective study by Chen et al. also compared the CA125 levels of BRCA1/2 mutation carriers to non-mutation carriers and found no significant difference between these groups. However, menopausal status was not considered as a confounder in the analysis, potentially causing misinterpretation of the results [40]. Our results further show a statistically significant interaction between BRCA1/2 mutation status and menopausal status on CA125 serum level. This means that the impact of menopausal status depended on the presence of a mutation in BRCA1/2. Accordingly, we observed a greater decrease of the CA125 serum level in postmenopausal mutation-carriers than in non-mutation-carriers.

Several studies have reported a significantly lower age at natural menopause in BRCA-mutation-carriers than in healthy non-carriers [41, 42], which could explain the greater decrease of the CA125 serum levels in BRCA-mutation-carriers, since they would have already been postmenopausal for a longer time. A study by Tea et al. even showed a significantly earlier onset of menopause in BRCA1-mutation carriers compared to BRCA2-mutation-carriers [43]. However, a recently performed meta-analysis, which combined the data of 1535 BRCA1/2-mutation-carriers and 3191 control individuals, did not support the hypothesis of an association between deleterious mutations in BRCA1/2 and an earlier onset of menopause [44]. Still, more carefully designed studies should be undertaken to resolve the question of how pathogenic germline mutations in BRCA1/2 and early menopause are truly associated, since various types of selection bias can influence the comparison of age at natural menopause between BRCA1/2-mutation-carriers and non-carriers.

The current approach to screening for ovarian cancer is based on the premise that the disease must be detected at stage I or II to increase chances of survival but is still impeded by low sensitivity and specificity [13, 15, 45]. According to our results and several studies reporting earlier onset of menopause in BRCA-mutation carriers [41–43], it can safely be assumed that lowering the CA125 cut-off value in postmenopausal BRCA-mutation-carriers would increase sensitivity for the detection of ovarian cancer in early stages. Nevertheless, it is important to keep in mind that there is an inherent tradeoff between sensitivity and specificity. Therefore, while some studies suggest lowering the CA125 threshold of 50 kU/L in postmenopausal women may enable us to detect more cancers at an earlier stage, it would also lead to an increased number of false positive results and overdiagnosis [12, 46].

Personalizing ovarian cancer screening by using an algorithm based on longitudinal CA125 information and more frequent testing has yielded promising results in high-risk women. For example, the risk of ovarian cancer algorithm (ROCA) detects significant elevations of each patient’s baseline CA125 level and thus increases the probability of earlier disease detection, even before the standard cut-off is reached, while specificity is maintained by excluding patients with high stable levels [26]. It has been suggested that including the BRCA mutation status in the mentioned algorithms and other risk-assessment models based on CA125 measurement, may help to further personalize and improve screening [10, 28]. However, due to the insufficient performance of serum CA125 measurement in large screening trials [47, 48], we do not anticipate this tumor marker becoming a validated part of ovarian cancer screening soon.

Our study has several limitations. Defining menopausal status solely based on age and without serum FSH and estradiol measurement has the potential to cause misinterpretation of the results. This is important to consider in patients with BRCA germline mutations and an increased risk of premature ovarian failure. Due to the retrospective character of this study, we were not able to consider the use of oral contraceptives, smoking and most of the comorbidities, which may influence CA125 levels in healthy women. Therefore, we had to exclude cases with values greater than 70 kU/L from our analysis. Additionally, we evaluated each measured CA125 serum value as its own case, meaning that we did not analyze the same amount of serum values in every patient. Considering that, as research suggests, each woman has her own CA125 baseline [26], the approach we took in our statistical analysis could prove to be problematic. This study is based on the data of women who participated in a high-risk early detection program for hereditary breast and ovarian cancer. Comparing BRCA1/2-mutation carriers with women who are not at high risk for breast or ovarian cancer, may have provided a stronger significance in our results.

## Conclusions

Our findings suggest that CA125 serum levels are subject to the same changes caused by ageing and menopause but behave differently in BRCA mutation carriers than in women without this mutation. Menopausal status remains the primary clinical factor affecting the CA125 serum level. We know of no underlying biological mechanism which could explain the more pronounced decrease of CA125 in BRCA1/2 mutation carriers with increasing age.

We currently lack studies evaluating CA125 screening in healthy BRCA1/2 mutation carriers. The available literature regarding this issue has been limited to small populations. Our cohort of 466 high-risk patients included 132 BRCA1/2 mutation carriers and 334 non-carriers, therefore representing one of the largest single-center study cohorts for high-risk patients to date. To our knowledge, this study is the first to describe serum CA125 levels in healthy BRCA1/2 mutation carriers in comparison to non-carriers, while also considering menopausal status. We anticipate that this study will soon inspire carefully designed prospective studies aiming to personalize CA125 screening in BRCA1/2 mutation carriers. The consequent results may lead to the definition of a new cut-off level of CA125 for women with high hereditary risk, especially in the postmenopausal setting and improve ovarian cancer screening.

## Data Availability

The datasets analyzed during the current study are available from the corresponding author upon reasonable request and with permission of the Medical University of Vienna.

## Abbreviations

ANOVA: Two-factor analysis of variance
CA125: Carbohydrate antigen 125
HBOC: Herditary breast and ovarian cancer
OC: Ovarian cancer
RRSO: Risk-reducing salpingo-oophorectomy
TVU: Transvaginal ultrasound
